# A gas-only bioreactor system maintains stable culture environments and reveals that moderate pH deviations trigger transcriptome-wide responses in human cells cultured in physioxia and physiological buffers

**DOI:** 10.1093/lifemedi/lnac056

**Published:** 2022-11-22

**Authors:** Silvia Arossa, Samhan M Alsolami, Shannon G Klein, Yingzi Zhang, Gerardo Ramos-Mandujano, Alexandra Steckbauer, Anieka J Parry, Juan Carlos Izpisúa-Belmonte, Carlos M Duarte, Mo Li

**Affiliations:** Red Sea Research Centre (RSRC) and Computational Bioscience Research Center (CBRC), King Abdullah University of Science and Technology, Thuwal 23955, Saudi Arabia; Bioscience Program, Biological and Environmental Science and Engineering Division (BESE), King Abdullah University of Science and Technology, Thuwal 23955, Saudi Arabia; Red Sea Research Centre (RSRC) and Computational Bioscience Research Center (CBRC), King Abdullah University of Science and Technology, Thuwal 23955, Saudi Arabia; Bioscience Program, Biological and Environmental Science and Engineering Division (BESE), King Abdullah University of Science and Technology, Thuwal 23955, Saudi Arabia; Bioscience Program, Biological and Environmental Science and Engineering Division (BESE), King Abdullah University of Science and Technology, Thuwal 23955, Saudi Arabia; Red Sea Research Centre (RSRC) and Computational Bioscience Research Center (CBRC), King Abdullah University of Science and Technology, Thuwal 23955, Saudi Arabia; Red Sea Research Centre (RSRC) and Computational Bioscience Research Center (CBRC), King Abdullah University of Science and Technology, Thuwal 23955, Saudi Arabia; Gene Expression Laboratory, Salk Institute for Biological Studies, 10010 North Torrey Pines Rd., La Jolla, CA 92037, USA; Altos Labs, Inc. 5510 Morehouse Drive, Suite 300, San Diego, CA 92121, USA; Red Sea Research Centre (RSRC) and Computational Bioscience Research Center (CBRC), King Abdullah University of Science and Technology, Thuwal 23955, Saudi Arabia; Bioscience Program, Biological and Environmental Science and Engineering Division (BESE), King Abdullah University of Science and Technology, Thuwal 23955, Saudi Arabia

## Dear editor,

Mammalian cells function optimally in a stable environment, where temperature, metabolic gases, and pH are controlled within tight margins *via* homeostasis. In human tissues, pH is maintained *via* the primary physiological buffer system, CO_2_/HCO_3_^−^ [1, 2]. However, exposure to excess atmospheric CO_2_ could decrease the ratio of bicarbonate to CO_2_, resulting in a shift towards a more acidic environment [3]. To understand the impact of this shift on human health, we must first examine how extracellular pH changes affect cellular performance. The acid–base balance of mammalian fluids is regulated by a suite of processes that maintain pH within a narrow range for optimal for cellular function (termed physiological pH) [4]. *In vitro*, extracellular pH is moderated by four processes; gas exchange at the atmosphere/media interface, media buffering capacity, partial pressure of carbon dioxide, and cellular metabolism [5]. Such processes operate simultaneously, making it difficult to control extracellular pH in cell culture within optimal ranges [6, 7]. Standardized techniques, including the use of exogenous buffers and CO_2_ incubators, that attempt to maintain physiologically relevant conditions during cell culture are commonly used. However, broad departures from CO_2_, O_2_, and pH setpoints are frequently encountered in routine experiments [6, 7], raising concerns over how such instabilities introduce experimental artifacts and affect reproducibility [2, 5]. Previous studies focusing on the effects of pH deviations on cellular performance typically use exogenous acid and/or bases (e.g., HCl, NaOH, non-volatile buffers) to adjust pH [2, 5], limiting the physiological relevance of the results obtained.

Here we developed a method, relying solely on the physiological buffer system, to address this research gap. We demonstrate that this method tightly maintains nominal pH levels within ± 0.013 units (SE) and sustains levels within physiological ranges (defined 7.35–7.45 for blood cells or physioxia) during prolonged cultures (hours to days). We utilized this approach to analyze the transcriptome of the human GM12878 cell line—a widely benchmarked cell line—cultured under different medium pH levels (6.8, 7.0, 7.2, and 7.4) during 72 h of culture.

**Figure 1.**
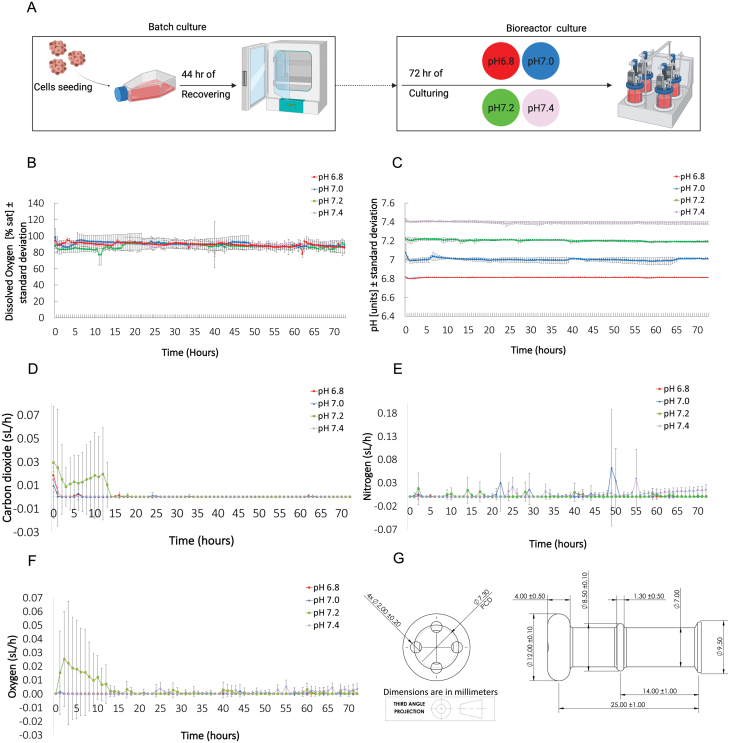
Maintaining pH and physioxia in a bioreactor using only gases and physiological buffers. (A) A schematic description of the experimental design. First, GM12878 cells (~2.0 × 10^5^ cells mL^−1^) were cultured under standard batch culture conditions for 44 h to allow for recovery from thawing. Cells were then transferred into a bioreactor system and exposed to four different pH levels for 72 h. Four independent trials were conducted per pH condition. (B) Average (± 1SD) *d*O_2_ levels at 30-min intervals over the 72-h experiment in the optimized bioreactor systems (*n* = 146). Mean *d*O_2_ (% air saturation) was 89.83% ± 5.78% across treatments (SD, *n* = 146; Fig. 1D). (C) Average (± 1SD) pH levels at 30-min intervals for the four pH treatments over the 72-h experiment in the optimized bioreactor systems (*n* = 146). pH levels were tightly controlled around the desired setpoints: 6.810 ± 0.003 (SD, *n* = 146), 7.000 ± 0.025 (SD, *n* = 146), 7.201 ± 0.012 (SD, *n* = 146), and 7.395 ± 0.013 (SD, *n* = 146) (Fig. 1C). (D–F) gas sparging rates (sL/h ± 1SD) during the 72-h experiment measured every hour. (G) Schematic representation of custom-made glass diffusers used to enhance the diffusion of gas in our experiments.

We modified the automatic control scripts and gas sparging system of the DASbox® Mini bioreactor system to maintain pH and physioxia using only gases and physiological buffers. Briefly, pH control was achieved by sparging CO_2_ or N_2_, when pH was higher or lower than the setpoint, respectively, in an automated feedback loop. Similarly, *d*O_2_ was stably maintained using pure O_2_ sparging (see Methods). These gases were delivered in a medium buffered solely with the physiological CO_2_/HCO_3_^−^ buffer system. GM12878 cultures were stably maintained at four different pH levels, ranging from the physiological pH 7.4 (pH 7.25–7.45 in mammalian arterial blood and irrigated tissues [4]) to 6.8, a value routinely observed in batch culture [5]. Across the four pH treatments, *d*O_2_ was constantly maintained at physiological *d*O_2_ levels (~85%) (Fig. 1A). The modified bioreactor setup permitted the stable control of temperature (37°C ± 0.001°C, *n* = 146), pH, and *d*O_2_ levels throughout the experiment (Fig. 1B and 1C). Custom-made diffusers were used to enhance rates of gas diffusion in the medium (Fig. 1G). The diffusers created microbubbles of the gases, which quicken the rate of gas/medium equilibration by increasing the contact area between the liquid medium and the gases. Gas sparging rates that maintained the setpoints are shown in Fig. 1D–F. Cell performance and transcriptome responses were examined every 24 h.

**Figure 2.**
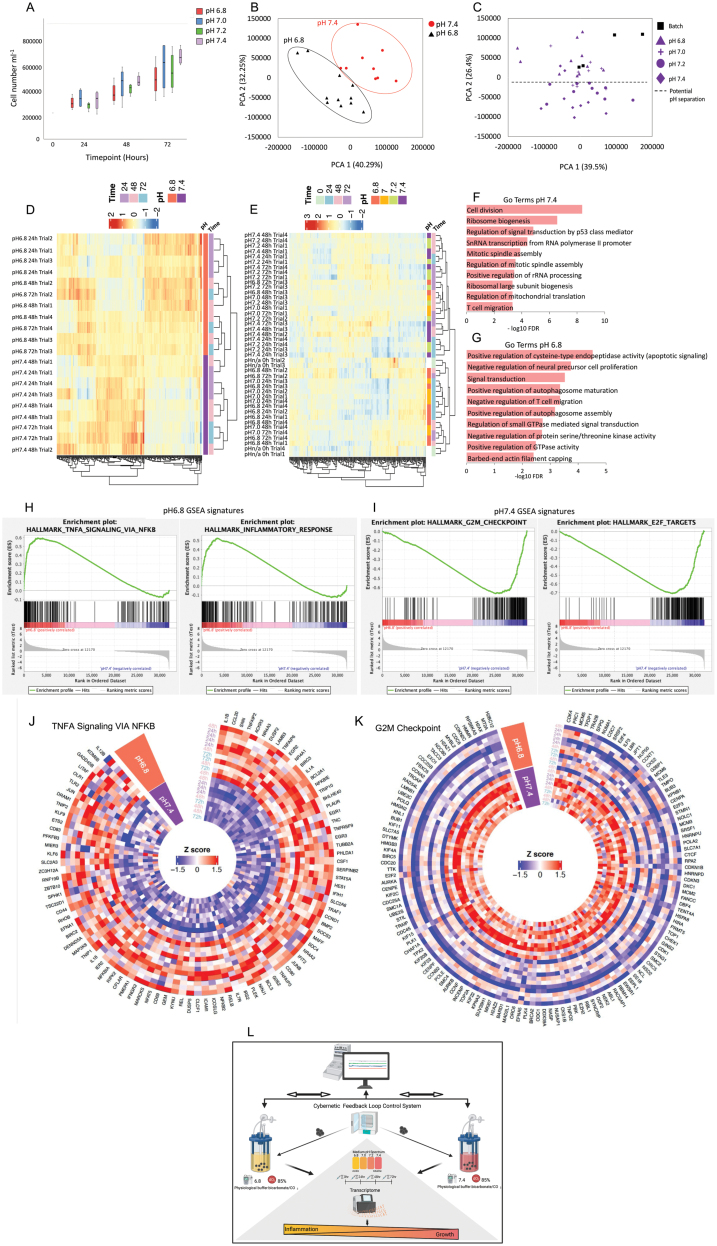
Effects of pH decline on cell performance and transcriptome. (A) Average cell abundance (live cells mL^−1^ ± SE) for each timepoint and for each pH treatment (biological replicates, *n* = 4). Initial cell viability and density were 87.00% ± 0.73% (mean ± 1SE) and ~2.0 × 10^5^ cells mL^−1^, respectively. The black line in the center of the boxes is the median and the bars represent the standard error (± 1 SE). The box represents the first and third quartiles. (B and C) PCA plots for RNA-Seq gene expression data of pH 6.8 and 7.4 (B) and RNA-seq data of all the pH levels (C). The first two principal components (PC1 and PC2) were plotted respectively on the *x*- and *y*-axis. The proportion of variance explained by each component is indicated next to the axis labels. Each dot represents one sampling (all time points in all trials are shown) and its color corresponds to the pH condition. (D and E) Heatmaps of variance stabilizing transformation (VST)-transformed values of the top 300 DEGs across the samples exposed to pH 6.8 and 7.4 (D) and across all samples and timepoints (E). Red-to-blue scale indicates the mean of VST-transformed values: red indicates gene expression above the average and blue indicates gene expression below the average. (F and G) GO term analysis performed on upregulated DEGs in pH 7.4 (F) and 6.8 (G) across all samples and timepoints (*N* = 4). The *x*-axis shows the −log_10_FDR values. (H and I) GSEA signatures enriched in cells cultured under pH 6.8 (H) and 7.4 (I) based on comparing transcriptomic data of pH 6.8 and 7.4 across all samples and timepoints (*N* = 4). The enrichment score is shown on the top *y*-axis, whereas the rank in ordered data is shown on the *x*-axis. (J and K) Heatmaps made using FPKM for TNFA signaling via NFKB GSEA signature genes (94 genes, J) or G2M checkpoint signature genes (137 genes, K) of pH 6.8 and 7.4 across all samples time points (*N* = 4). Color scale indicates the Z score: red indicates genes that are expressed above the average and blue indicates genes that are expressed below the average. (L) Graphical abstract of the study.

Cell viability did not differ among the pH treatments and was consistent over time (81.51% ± 0.79%; *P* > 0.05). As expected, cell density increased over time (*P* < 0.001, Fig. 2A) and was unaffected by the pH treatments. However, there was a significant interaction between the factors of pH and time on growth rates, which resulted from inconsistent rates among the pH treatments over time (*P* < 0.05).

Our system provided a unique opportunity to assess cellular responses under different magnitudes of pH decline, where pH and *d*O_2_ were continuously monitored and tightly controlled. We thus performed RNA-seq of GM12878 cells at 24-h intervals in four independent trials (see Methods). A principal component analysis (PCA) of RNA-seq data revealed a marked separation of the pH 6.8 and 7.4 clusters in the PC1 and PC2 dimensions (Fig. 2B). Similar pH-driven transcriptomic differences in PC2 were similarly observed across all pH treatments, and across other factors (e.g., sampling time point, independent trial) that equally affected all conditions (PC1, Fig. 2C). Overall, the transcriptome of cells cultured under pH 7.4 (and pH 7.2 to a lesser extent) differed from that of the initial batch culture and also differed from cells under pH 6.8 and 7.0. The data suggested distinct transcriptome profiles between cells grown in physiological pH and those that experienced low pH, for which cells routinely encounter during published batch culture experiments. Unsupervised hierarchical clustering between pH 6.8 and 7.4 further highlighted differences between the gene expression of the two most disparate pH treatments (Fig. 2D). Similar clustering of gene expression profiles by pH was also observed in across all conditions (Fig. 2E).

Differential gene expression analysis between pH 7.4 and 6.8 was performed on 48,557 annotated genes and identified 3897 differentially expressed genes (DEGs) (FDR < 0.05). 1985 genes were upregulated in pH 6.8 relative to the pH 7.4 treatment and 1912 genes were downregulated. Gene Ontology (GO) term enrichment analysis of upregulated DEGs (FDR < 0.05) highlighted striking differences between biological processes of cells grown at pH 7.4 and 6.8. The top ten most enriched GO terms among the upregulated genes in pH 7.4 included cellular division and ribosome biogenesis (Fig. 2F). This was in stark contrast to GO terms enriched in upregulated genes in pH 6.8, which included apoptotic signaling pathways and negative regulation of cellular proliferation (Fig. 2G). Additionally, Gene Set Enrichment Analysis (GSEA) performed on all gene expression data of pH 6.8 and 7.4 across all sampling time points uncovered significant inflammation signatures at pH 6.8 (Fig. 2H), and significant cell-cycle signatures at pH 7.4 (Fig. 2I). Enriched genes in the TNFA signaling via NFKB and the G2M checkpoint signatures were used to construct heatmaps. Genes associated with inflammation such as IL1B, NFKBIA, and RELB, all well-known mediator of cellular inflammatory responses, were consistently upregulated in the pH 6.8 cultures (Fig. 2J). Similarly, the G2M checkpoint signature genes including CDK4, CDK1, and CDC6 were among the most highly upregulated genes in the pH 7.4 samples (Fig. 2K).

To the best of our knowledge, our modified bioreactor is the first system that allows the cellular environment to be continuously monitored and tightly controlled using only gas delivery and the bicarbonate/CO_2_ buffer system. Specifically, we show that cybernetic manipulation of pure CO_2_, O_2_, and N_2_ gases can be used to simultaneously and stably maintain pH and *d*O_2_ in a physiologically relevant bicarbonate buffer system. Additionally, we demonstrate that deviations of medium pH from physiological values significantly impact the transcriptome of the GM12878 cell line. On closer inspection, we observed that gene expression patterns increasingly drift away from that of the physiological pH, as pH decreases. Our analysis of affected biological processes revealed that inflammation- and apoptosis-related genes were significantly upregulated in low pH (e.g., pH 6.8). In contrast, processes involved in cell proliferation were upregulated at physiological pH levels (e.g., pH 7.4), which agreed with cell growth data.

Only one study, to the best of our knowledge, has reported the sole use of gas to control pH in cell culture [8]. However, *d*O_2_ levels were not reported and the study acknowledged unexpected *d*O_2_ fluctuations, leading to uncertainties over the interpretation of the results. It is likely that the control loop for pH used in this study (CO_2_/air-N_2_) would have caused departures in *d*O_2_ from the setpoint. We used pure N_2_ and CO_2_ delivery to increase and decrease pH, respectively, and the sparging of pure O_2_ to correct *d*O_2_ levels. Rates of CO_2_ sparging were highest at the beginning of the experiment because the initial medium pH deviated from the set-point (Fig. 1D). Beyond this point, cellular metabolism continuously acidifies the medium, thereby reducing the need for a negative pH control (i.e., CO_2_ sparging). Whereas, N_2_ sparging is required for displacing CO_2_ when pH is below the setpoint. This explains the fluctuations of injected N_2_ during the experiment and the pattern observed in CO_2_ sparging rates in Fig. 1D–F. Although some fluctuations were observed in the O_2_ sparging rates, especially when initial medium conditions deviated from the setpoint, our method kept O_2_ levels close to the setpoint and the magnitude of fluctuations were similar across the pH treatments. Given this, we expect the effects of such fluctuations on cellular responses to be marginal and similar across the pH treatments tested, although future research is required to further refine *d*O_2_ control.

Our bioreactor method offers precise and physiologically relevant controls over pH and other environmental factors. Hence, this approach could improve the power of discovery of future research on the biological significance of cellular environment. This method might also find applicability in clinical-grade expansion of stem cells [e.g., pluripotent stem cells (PSCs) and hematopoietic stem cells] in already existing gas-based bioreactor systems. By relying on a feedback control loop for controlling environmental conditions, this bioreactor protocol could also be used with cells that quickly acidify the medium (e.g., cancer cells) and to support long-term cultures independent of batch culture. The gene signatures that resulted from comparisons of such stringently controlled cultures may facilitate mechanistic studies on how pH variations contribute to inflammation and metabolism as recently reported in the immune system [9]. For instance, functional studies on migration and metabolism could be prioritized.

## Research limitations

This work is limited to developing a bioreactor platform that controls pH and *d*O_2_ in a physiologically relevant manner, and to an initial analysis of transcriptomic responses to different pH levels. Functional assays to further elucidate the meaning of the observed changes in gene expression are underway. We used a common cell line model to demonstrate the utility of the developed gas-only bioreactor. Future studies should consider adapting this new system to support the culture of various cell types (e.g., PSCs and cancer cells).

## Data availability

The bulk RNA-seq data reported in this paper are deposited in gene expression omnibus with the following accession code, GEO: GSE203180. Deposited GEO data can be accessed with token ubovkaiwhrutdar. Raw data about environmental conditions and cell performance are provided as supplementary materials.

## Supplementary Material

lnac056_suppl_Supplementary_Methods

lnac056_suppl_Supplementary_Materials
