# Supplementary material for: A gas-only bioreactor system maintains stable culture environments and reveals that moderate pH deviations trigger transcriptome-wide responses in human cells cultured in physioxia and physiological buffers
Source: Life Med. 2022 Nov 22;1(2):236–40. doi: 10.1093/lifemedi/lnac056 (PMC11749540; doi:10.1093/lifemedi/lnac056)
Supplement: lnac056_suppl_Supplementary_Methods [file lnac056_suppl_Supplementary_Methods.docx]

Materials and methods

*Cell culture*

Human lymphoblastoid cells (GM12878 cell line; passage 6; 200,000 cells mL^−1^ as initial seeding density; Coriell Institute) were expanded in HERAcell 150i Thermo Scientific incubators (conditions set at 37°C, 90% humidity and 5% CO_2_) in T175 flasks (Gibco, identifier 734-0050) in 50 mL of RPMI-1640 medium (Thermo Fisher, identifier 31800105), which employed the physiological HCO_3_^−^/CO_2_ buffer system (Sodium bicarbonate by Gibco, identifier 25080-102). These expanded cells were harvested, placed in Mr Frosty^TM^ (ThermoFisher Scientific), temporarily transitioned into a −80°C freezer, and then transferred into an N_2_ tank for long-term storage. Prior to each experiment, cells were thawed in batch culture and allowed 44 h to recover before subsequent experiments. All reagents and consumables used in this study can be found in Table S1.

*Experimental design*

The experimental design consisted of one factor (pH) with four levels (pH 7.4 vs. 7.2 vs. 7.0 vs. 6.8). GM12878 cells were cultured under the four different pH levels for 72 h within autoclavable glass bioreactor vessels connected to a commercial bioreactor system (4-fold, max. 5 sL/h gassing, DASBox® Mini Bioreactor system controlled by the DASware® Software Suite). Since the bioreactor system set-up permitted only four bioreactor vessels to be controlled simultaneously, we conducted four consecutive experiments to obtain sufficient replication (*n* = 4) for each pH level. For each experiment, one bioreactor unit was allocated to each pH level (i.e., *n* = 1 per pH). To minimize potential bias associated with individual bioreactor vessels, each bioreactor vessel (and control unit) was allocated a different pH level in each experiment.

Prior to the start of the experiment, pH sensors of each bioreactor vessel were calibrated using a two-point calibration (NIST traceable buffers at pH 7 and calibration slope of pH 4 as recommended by the manufacturer, Orion™). Then, the four vessels were assembled and 100 mL of PBS (−/−) (Gibco, identifier 14190144) was added and autoclaved at 120°C for 20 min as recommended by the manufacturer. The vessels were then placed in a clean pre-sterilized safety cabinet for ~1 h and cooled to room temperature. *d*O_2_ sensors (Hamilton®, identifier 76DXPHHMC120) were calibrated after autoclaving in PBS (−/−) using a two-point calibration (i.e., 100% and 0% air saturation) and then polarized overnight. After calibration, the vessels were disconnected from the bioreactor system, externally sterilized with Ethanol 70%, and placed into a clean and pre-sterilized culture hood for the removal of PBS. 140 mL of the RPMI-1640 medium was then added to each bioreactor vessel using aseptic techniques and equilibrated overnight to the desired medium chemistry. After the equilibration took place, the cells were inoculated into each bioreactor vessel in 10 mL of medium, equating to a final concentration of 200,000 cells mL^−1^ in a total 150 mL working volume. Throughout experimentation, the cells were routinely tested for mycoplasma using a Lonza Mycoalert kit (Lonza, identifier LT07-118) and observed under an inverted microscope (at 20× magnification) for eventual sign of bacterial contamination.

*Manipulation of culture medium chemistry*

To achieve the desired medium chemistry of each treatment, a DASBox® Mini Bioreactor system (Eppendorf, identifier OT30QUOTE) controlled by the DASware® Software Suite was used to deliver CO_2_, N_2_, and O_2_ gas to four bioreactor units (model: 76DS0250ODSS, Eppendorf). The integrated mass-flow controller system manipulated pH (and the HCO_3_^−^/CO_2_ buffer system) in each of the four treatments (pH levels: 7.4, 7.2, 7.0, and 6.8) via a two-sided feedback loop, whereby deviations in pH above or below the setpoint detected by pH sensors (Hamilton®, identifier 76DXPHHMC120) would trigger the addition of gas. Specifically, CO_2_ gas was used to lower pH (via the formation of carbonic acid) and N_2_ gas was used to displace CO_2_, thereby increasing medium pH. Additionally, the sparging tube was equipped with custom made autoclavable glass diffusers, which eased the diffusion of gases into the medium. Although the DASbox® Mini Bioreactor system is designed to run two-sided pH control using either acid/base or CO_2_/base control, we re-configured the control loop to use CO_2_/N_2_. CO_2_/N_2_ was specifically chosen so that reductions in pH reflected increases in CO_2_ equilibria. Dissolved O_2_ levels were held constant across the four pH treatments at ~85% air saturation using a one-sided control loop employing pure O_2_. When variations in *d*O_2_ levels were detected by the O_2_ sensor, pure O_2_ delivery was triggered. The temperature was set at 37°C (Platinum RTS temperature sensor, Eppendorf, identifier 78103308) and agitation speed was set at 155 rpm (8-Blade impeller, 60°pitch, Eppendorf, identifier 78107377). Temperature, dO_2_ and pH levels were monitored at 30 min intervals throughout the experiment.

*Gene expression analysis, cell counts, and growth rate calculation*

An aliquot of 1 mL of cell suspension was sampled at 24, 48, and 72 h from each of the four replicate vessels within each pH treatment (i.e., *n* =16) for cell performance analysis and RNA extraction. Cell density and viability was measured in duplicate in a volume of 10 µL using a Countess II Automated cell counter (Thermo Fisher, identifier AMQAF1000). Cell growth was calculated as % day^−1^. One million GM12878 cells were sub-sampled from the aforementioned aliquotes for RNA extraction and subsequent analyses. The cells were placed in RNAlater™ Stabilization Solution (Invitrogen, identifier AM7021) and stored in −80°C. RNA was then extracted using Qiagen RNeasy micro kit (Qiagen, identifier 74004) at the same time for all the samples, according to manufacturer recommendations. RNA quality was assessed using a Bioanalyzer 2100 system (Agilent, identifier G2939BA) with a nanochip. RNA concentration was determined using a Qubit 4 fluorometer (Thermo Fisher, identifier Q33226). RNA samples characterized by an RNA Integrity Number having >7 were outsourced to Novogene for sequencing, and the library was prepared following a strand-specific approach.

*Data analysis and statistics*

*Differential gene expression analyses*

The FASTQC files were used as input for the analyses performed in A.I.R., an online tool provided by Sequentia Biotech. A total of 48,557 genes of which 13,937 genes passed HTS filter (i.e., pH 6.8 and 7.4). The EdgeR pipeline was run on these data, thus obtaining PCA and GO terms analyses via the A.I.R. online tool. The heatmaps (Fig. 2D and 2E) were constructed using R (version 4.1.1) utilizing DESeq2 (version 1.32.0) to perform differential expression analysis with vst () function to perform the variance stabilizing transformation as well as using genefilter (version 1.7.4.1) to filter out the top 300 genes with the highest variance across the samples. Pheatmap (version 1.0.12) was used to obtain the heatmaps. Furthermore, the signature derived heatmaps (Fig. 2H and 2I) were made using pheatmap. Finally, GSEA (version 4.1.0) analysis with MSigDB.v7.4 chip with default settings was performed on the entire FPKM normalized RNA-seq dataset for pH 6.8 and 7.4 using all time points.

*Statistical analysis of cell count, viability, and growth rate*

The dependent variables (cell count, viability, and growth rate) were analyzed using repeated Linear mixed models (LMMs) in SPSS. Prior to the analysis, all dependent variables were tested for normality and homoscedasticity using Shapiro–Wilk’s tests, standardized residual plots, and Q–Q plots and if required, data were log-transformed. In this case, cell viability and live cell count were normally distributed, therefore no transformation was applied. Whereas, cell growth was not normally distributed and a log-transformation was necessary to meet normality assumptions. For each response variable, the fixed factors were the four-pH treatments (6.8, 7.0, 7.2, and 7.4) and time (0, 24, 48, and 72 h). Time was also the repeated measure. To test for potential bias associated with individual bioreactor units (Unit 1–4) and/or experiments (Trials 1–4), we included two random terms (or blocks) in all LMMs analyses: “Bioreactor unit (pH × time)” and “Batch (pH × Time)”. The Wald Z test of simultaneous coefficients and estimated covariance parameters were used to assess whether the random terms significantly affected the models. If the random terms were found to be redundant, they were removed, and the analysis rerun. We compared various goodness-of-fit statistics (e.g., Bayesian information criterion, Akaike’s information criterion, and −2 restricted log likelihood) to investigate numerous repeated covariance structures [e.g., Compound Symmetry (CS) and Autoregressive (AR) [1] and identify the model-of-best fit. For our data, the repeated covariance structures were the following: CS for cell viability and cell growth, and AR [1] for live cell count. Preliminary analyses for all three dependent variables revealed that the random terms accounting for individual bioreactor units and/or experiment batches did not affect the dependent variables. Hence, the random terms were removed, and the analyses rerun. If significant effects were found, post hoc comparisons of least-squares means (estimated marginal means) were conducted to identify where differences occurred for the significant, highest-order terms. In the graphs, variables were grouped by significant variables as a result of LMMs analyses.
