# Supplementary material for: A gas-only bioreactor system maintains stable culture environments and reveals that moderate pH deviations trigger transcriptome-wide responses in human cells cultured in physioxia and physiological buffers
Source: Life Med. 2022 Nov 22;1(2):236–40. doi: 10.1093/lifemedi/lnac056 (PMC11749540; doi:10.1093/lifemedi/lnac056)
Supplement: lnac056_suppl_Supplementary_Materials [file lnac056_suppl_Supplementary_Materials.docx]

**Moderate pH deviations Trigger Transcriptome-wide Responses in Human Cells Cultured in Physioxia and Physiological Buffers**

Silvia Arossa^1,$^, Samhan M. Alsolami^2,$^, Shannon G. Klein^1^, Alexandra Steckbauer^1^, Anieka J. Parry^1^, Yingzi Zhang^2^, Gerardo Ramos-Mandujano^2^, Juan Carlos Izpisua-Belmonte^3,^*, Carlos M. Duarte^1,^*, and Mo Li^2,^*

**Table 1.** Key Resources Table indicates the source of the materials and consumables used in this study.

| **REAGENT or RESOURCE** | **SOURCE** | **IDENTIFIER** |
| --- | --- | --- |
| **Reagents** |  |  |
| RPMI-1640 | Thermo Fisher | 31800105 |
| FBS | Gibco | 26140-079 |
| PBS (-/-) | Gibco | 14190144 |
| Pen/Strep | Gibco | 15070-063 |
| T75 flasks | VWR | 734-0050 |
| Sodium bicarbonate (NaHCO_3_^-^) | Gibco | 25080-102 |
| Qiagen RNeasy kit | Qiagen | 74004 |
| MycoAlert | Lonza | LT07-118 |
| RNAlater™ stabilization solution | Invitrogen | AM7021 |
| NIST traceable buffer solutions (pH 7) | Orion™ | ORIO910760 |
| NIST traceable buffer solutions (pH 4) | Orion™ |  |
| O_2_ (gas) |  |  |
| CO_2_ (gas) |  |  |
| N_2_ (gas) |  |  |
| Extra pure water (for Diffusers) |  |  |
| **Equipment** |  |  |
| Hera i150 incubator | Thermo Scientific | 50116047 |
| DASbox Mini Bioreactor | Eppendorf | OT30QUOTE |
| DASbox 250 mL vessel | Eppendorf | DS0250ODSS |
| pH Sensor | Hamilton® | 76DXPHHMC120 |
| DO Sensor | Hamilton® | 76DXPOHMC120 |
| 8-Blade impeller, 60°pitch | Eppendorf | 78107377 |
| Platinum RTS temperature sensor | Eppendorf | 78103308 |
| Table top pH meter | Thermo Fisher | 2115001 |
| Countess II Automated cell counter | Thermo Fisher | AMQAF1000 |
| Bioanalzyer 2100 | Agilent | G2939BA |
| Inverted microscope | Olympus | CKX53 |
| Qubit 4 | Thermo fisher | Q33226 |
| Glass diffusers with o-ring | Manufactured by KAUST Core Labs | N/A |
| **Software or platform** |  |  |
| DASware ® Software | Sequentia biotech | N/A |
| R version | N/A | version 3.6.3 |
| **Cell lines** | Eppendorf | N/A |
| GM12878 | Corriel | N/A |
